# Caregivers’ burden of care during emergency department care transitions among older adults: a mixed methods cohort study

**DOI:** 10.1186/s12877-024-05388-1

**Published:** 2024-09-28

**Authors:** Nathalie Germain, Estephanie Jémus-Gonzalez, Vanessa Couture, Émilie Côté, Michèle Morin, Annie Toulouse-Fournier, Laetitia Bert, Raphaëlle Giguère, Samir Sinha, Nadia Sourial, Lucas B. Chartier, Holly O. Witteman, France Légaré, Rawane Samb, Stéphane Turcotte, Sam Chandavong, Lyna Abrougui, Joanie Robitaille, Patrick M. Archambault, on behalf of the Network of Canadian Emergency Researchers, Patrick M. Archambault

**Affiliations:** 1 Centre de Recherche Intégrée Pour Un Système Apprenant en Santé Et Services Sociaux, Centre Intégré de Santé Et Services Sociaux de Chaudière-Appalaches, Lévis, Québec Canada; 2 Faculty of Medicine, Université Laval, Québec, Québec Canada; 3 VITAM - Centre de Recherche en Santé Durable, Québec, Québec Canada; 4 Faculty of Science and Engineering, Université Laval, Québec, Québec Canada; 5 Department of Family and Community Medicine, University of Toronto, Toronto, ON Canada; 6 Department of Medicine, University of Toronto, Toronto, Canada; 7 Department of Medicine, Sinai Health System and University Health Network, Toronto, ON Canada; 8 Department of Health Management, Evaluation and Policy, School of Public Health, Université de Montréal, Montréal, Québec Canada; 9 Department of Emergency Medicine, University Health Network, Toronto, ON Canada; 10 Department of Family Medicine and Emergency Medicine, Université Laval, Québec, Québec Canada; 11 Centre de Recherche du CHU de Québec - Université Laval, Axe Santé Des Populations Et Pratiques Optimales en Santé, Université Laval, Québec, Québec Canada; 12 Canadian Association of Emergency Physicians, Ottawa, ON Canada

**Keywords:** Older adult patients, Caregiving, Emergency care, Care transitions, Caregiver perceptions

## Abstract

**Objective:**

Improving care transitions for older adults can reduce emergency department (ED) revisits, and the strain placed upon caregivers. We analyzed whether caregivers felt a change in burden following a care transition, and what may be improved to reduce it.

**Methods:**

This mixed-methods observational study nested within LEARNING WISDOM included caregivers of older patients who experienced an ED care transition. Burden was collected with the brief Zarit Burden Interview (ZBI-12), and caregivers also commented on the care transition. A qualitative coding scheme of patient care transitions was created to reflect themes important to caregivers. Comments were randomly analyzed until saturation and themes were extracted from the data. We followed both the SRQR and STROBE checklists.

**Results:**

Comments from 581 caregivers (mean age (SD) 64.5 (12.3), 68% women) caring for patients (mean age (SD) 77.2 (7.54), 48% women) were analyzed. Caregivers overwhelmingly reported dissatisfaction and unmet service expectations, particularly with home care and domestic help. Communication and follow-up from the ED emerged as an area for improvement. Caregivers who reported an increased level of burden following a patient’s care transition had significantly higher ZBI scores than caregivers who self-reported stable burden levels.

**Conclusion:**

Caregivers with increasing, stable, and improved levels of subjective burden all reported areas for improvement in the care transition process. Themes centering on the capacity to live at home and inadequate communication were most frequently mentioned and may represent serious challenges to caregivers. Addressing these challenges could improve both caregiver burden and ED care transitions.

**Supplementary Information:**

The online version contains supplementary material available at 10.1186/s12877-024-05388-1.

## Key points

1. We analyzed caregivers’ thoughts about emergency department care transitions using both qualitative and quantitative tools.

2. Caregivers reported dissatisfaction and unmet service expectations with home care, domestic help, and coordinating follow-ups.

3. Variance in self-reported subjective caregiver burden corresponds to Zarit Burden Interview (ZBI) scores.

**Supplementary Information:**

The online version contains supplementary material available at 10.1186/s12877-024-05388-1.

## Background

Populations around the world are undergoing a significant demographic shift, marked by the steady growth of older adults as a segment of society, and the shrinking of the available workforce of those who care for older people [1]. Within this context, informal caregivers are being increasingly tasked to fill in this gap [2]. Most care is now handled by caregivers, from activities of daily living to minor medical procedures [3]. Caregivers help older adults adhere to treatment plans and medication schedules [4], remain in their communities [5], and avoid premature long-term care [6], while also playing a critical role in the passage of patients between levels of health care and across care settings, commonly known as care transitions.

One such care transition that frequently affects older patients and their caregivers occurs when a patient receives medical attention at the emergency department (ED) and is then discharged home. An ED visit by a community-dwelling older adult often signals a significant shift in their health status and can signal the beginning of a decline in their independence [7]. Caregiving during an episode requiring emergency care may present additional challenges and can exact a toll on caregivers both physically and emotionally. This confluence of challenges is commonly referred to as caregiver burden [8, 9]. Burden can arise from the intensity and duration of caregiving responsibilities, a lack of support or coping mechanisms, and the condition of the patient [10]. High levels of caregiver burden may predispose caregivers to burnout, thereby impairing their capacity to provide effective care [11]. The health of patients and their caregivers may deteriorate if their caregiver is overwhelmed or incapacitated, resulting in poor outpatient clinical outcomes and an increase in avoidable ED revisits [12, 13].

For emergency clinicians, addressing caregiver burden often extends beyond what is possible during a brief assessment and initial treatment in the ED. Caregivers in situations involving ED care for patients and experiencing a discharge back home from the ED have previously reported that discharge plans are often drawn by healthcare providers that depend on the caregiver—without consulting them as to the feasibility of the plan [14, 15]. Patients may also decline professional home care services like bathing or administering medications, preferring having their caregiver perform these tasks. This may be to the detriment of the caregiver, who may not be comfortable taking on that role [14, 15].

Questionnaires like the Zarit Burden Interview (ZBI) exist to screen for caregivers experiencing caregiver burden. About 40% of caregivers experience a high level of caregiver burden when older patients seek acute medical care at the ED [16]. Little is still known about how the experience of having a patient seek acute care at the ED may worsen or improve an existing level of caregiver burden, or what can be done to improve it following an ED care transition. The purpose of this concurrent mixed methods [17] descriptive study is to analyze caregiver burden from several angles: quantitatively using the ZBI, ordinally by identifying a level of change following an ED visit, and qualitatively by identifying sources of caregiver burden. We also broach potential policy and practice changes necessary to alleviate the sources of caregiver burden.

## Method

### Study design and context

This study was nested within the longitudinal cohort study of an integrated health research project within the *Centre intégré de santé et de services sociaux de Chaudière-Appalaches* (CISSS-CA): LEARNING WISDOM (*Supporting the Creation of a LEARNing INteGrated Health System to Mobilize Context-adapted Knowledge with a Wiki Platform to Improve the Transitions of Frail Seniors From Hospitals and Emergency Departments to the cOMmunity*) [18]. The LEARNING WISDOM cohort included older adults and their caregivers who underwent a transition of care following a visit to one of four EDs in the CISSS-CA between January 2019 and December 2021. The CISSS-CA is an integrated health organization consisting of four acute care hospitals: the Hôtel-Dieu de Lévis (HDL), Hôpital de Saint-Georges (HSG), Hôpital de Montmagny (HDM), and Hôpital de Thetford Mines (HDM). HDL is a university teaching hospital receiving more than 78,000 annual ED visits while the other three rural sites each receive more than 35,000 visits.

The protocol for this study was approved by the CISSS-CA Ethics Review Committee (project #2018–462, 2018–007). We adhered to the Standards for Reporting Qualitative Research (SRQR) [19] guidelines for the assessment of qualitative outcomes and employed The Strengthening the Reporting of Observational Studies in Epidemiology (STROBE) Statement [20] to report the quantitative outcomes.

### Participants

LEARNING WISDOM [18] included consenting patients aged 65 years or older, who had been discharged back to the community from the ED observation unit. Patients only seen in the ambulatory care section of the ED, admitted to hospital, transferred to another hospital, or transferred to a long-term care center were excluded. Patients and their caregivers had to understand and speak French. For the full duration of the study recruitment period, at each participating hospital, a list of eligible discharged patients was generated each day. Patient phone numbers were selected using a computer-generated daily randomized list and patients were contacted to participate. No additional eligibility criteria were added for this specific study.

### Data collection

Using a deductive approach with an a-priori coding scheme developed in a previous study of patient comments [21], we designed this mixed-methods descriptive study to analyze data collected directly from the caregivers of older patients having experienced a care transition. As part of a continuous quality improvement project led by the CISSS-CA, patients were contacted by telephone between 24 h to 7 days after ED discharge. Patients were then subsequently invited to participate in a research project in the following days. We used the Nova Scotia Criteria to establish informed consent among patients [21, 22]. Patients were then asked if they consented to have their caregivers contacted by the research team. Demographic characteristics for patients and caregivers were collected using a structured interview, while some patient characteristics (e.g., comorbidities) were collected with chart review.

Informed consent was then also obtained for all contacted caregivers, who were then administered the Zarit Burden Interview (ZBI). The ZBI is the most widely used instrument measuring caregiver burden [23]. The reliability of scores on the ZBI measured by internal consistency (Cronbach’s alpha) is high, between 0.84–0.93 [24]. We used a short French Canadian version of the ZBI [9] consisting of 12 items with two constructs: role strain (items 1–9) and personal strain (10–12). Each question is scored by frequency in a five-point Likert scale (0 to 4): 0 for never, 1 for rarely, 2 for sometimes, 3 for quite often, and 4 for all the time. The scores are then summed into an overall indication of burden (range 0–48). For caregivers of patients with a major neurocognitive disorder, < 3 is categorized as low burden, 3–8 as moderate, 9–18 as high, and > 18 as severe [9].

Caregivers also answered two open-ended questions in as much or as little detail as they wished. Translated from the original French, the first one (Question A) was: “*In your opinion, has there been a change in the burden of care following [the patient’s] departure from the emergency department*?”. The second (Question B) was: “*In your opinion, what could be improved to reduce the burden of care for [the patient]?*”. Research professionals recorded critical elements of each patient’s response with important verbatim excerpts with text in a REDCap (Research Electronic Data Capture) [25, 26] database. These professionals (research nurses, and two PhD psychology students) were trained by the research team and authorized by the Director of Nursing and the Professional Services Director to perform data collection.

Responses to Question A were classified according to four ordinal categories: burden increased, burden decreased, burden unchanged, burden improved, and no comment. Inter-rater reliability of the coding of these categories was calculated with Cohen’s Kappa [27].

We used a concurrent mixed deductive approach for content analysis, in which we sought to qualify and quantify the themes present in responses to Question B [28]. We first used a hypothetico-deductive framework, in which we used an existing model of patient experiences of care transitions [21] to capture and systematically analyze the perspectives of caregivers as told through open-ended response data [29, 30]. The original coding framework for patient comments included 4 main themes (*Care in the emergency department*, *Conditions of stay*, *Independent living at home*, and *Discharge*) 19 sub-themes [21]. Changes made to this coding framework to better reflect caregiver experiences are discussed in the Results.

For coding, we noted when a sub-theme appeared in a comment (1 for affirmative, 0 for no mention) in addition to its emotional valence, which reflects the extent to which a comment reads as positive or negative in its statement. We used a quantifiable metric scaling system in which we rate the emotional valence of the comment: 0 negative, 1 positive, and 2 neutral. Importantly, we coded the absence of a requested service or item as negative because we argue that unmet needs are negative in valence.

Inter-rater reliability was established by independently coding 40 randomly selected comments (Responses to Question B) in parallel. The resulting reliability coefficient was high (Krippendorff’s Alpha: 0.90) [31]. Disagreements were then resolved by discussion between the two coders (NG and EJG) and the principal investigator (PA). The analysis was performed by two female evaluators from different scientific backgrounds (NG, an MSc student in epidemiology with training in mixed methods, and EJG, an MD student with research and clinical experience) and supervised by an experienced clinician researcher with expertise in qualitative analyses (PA) [32].

It was planned that after coding comments using this patient-centered coding scheme [21] and establishing inter-rater reliability, we would shift the model from patient-centered to caregiver-centered using an inductive/deductive hybrid thematic approach [33]. We allowed for new themes to emerge, and themes that did not fit with the data to be dropped. We then systematically applied this amended model of caregiver experiences to code the dataset. At this stage, two coders (NG and EJG) performed content analysis until saturation, stopping when additional comments did not reveal new themes [34]. Each individually coded 30 randomly selected comments per hospital (selection without replacement) [34], then additional randomly selected comments in rounds of 10. Saturation was achieved when coding 2 consecutive rounds of 10 without the emergence of a new theme per hospital.

### Statistical and visual analyses

An a-priori power analysis was conducted for LEARNING WISDOM and is described elsewhere [18]. No a-priori power analysis was conducted for the analyses in this article. For caregivers included in content analysis, we conducted a binomial test of the proportion of each self-reported burden change category versus chance. If the groups were distributed randomly, and there was no pattern of changes in subjective burden following a care transition, we would expect each category to contain 25% of caregivers.

To corroborate scores on the ZBI with caregiver reports as to how their level of burden may have increased. We conducted a one-way analysis of variance (ANOVA) of subjective change in burden on ZBI score. The assumption of homogeneity of variance was violated (Levene’s test *F*(3, 577) = 3.18, *p* = 0.023), so we conducted the ANOVA with a Brown-Forsythe correction. We also conducted a one-way analysis of covariance (ANCOVA) to determine if a difference on ZBI scores exists as a function of their self-reported change in subjective burden while controlling for age and comorbidities.

For comments containing two or more themes, we used the *quanteda* package in R (Quantitative Analysis of Textual Data) [35] to visualize and organize concurrent themes within caregiver comments using a series of co-occurrence network plots.

## Results

**Fig. 1:**
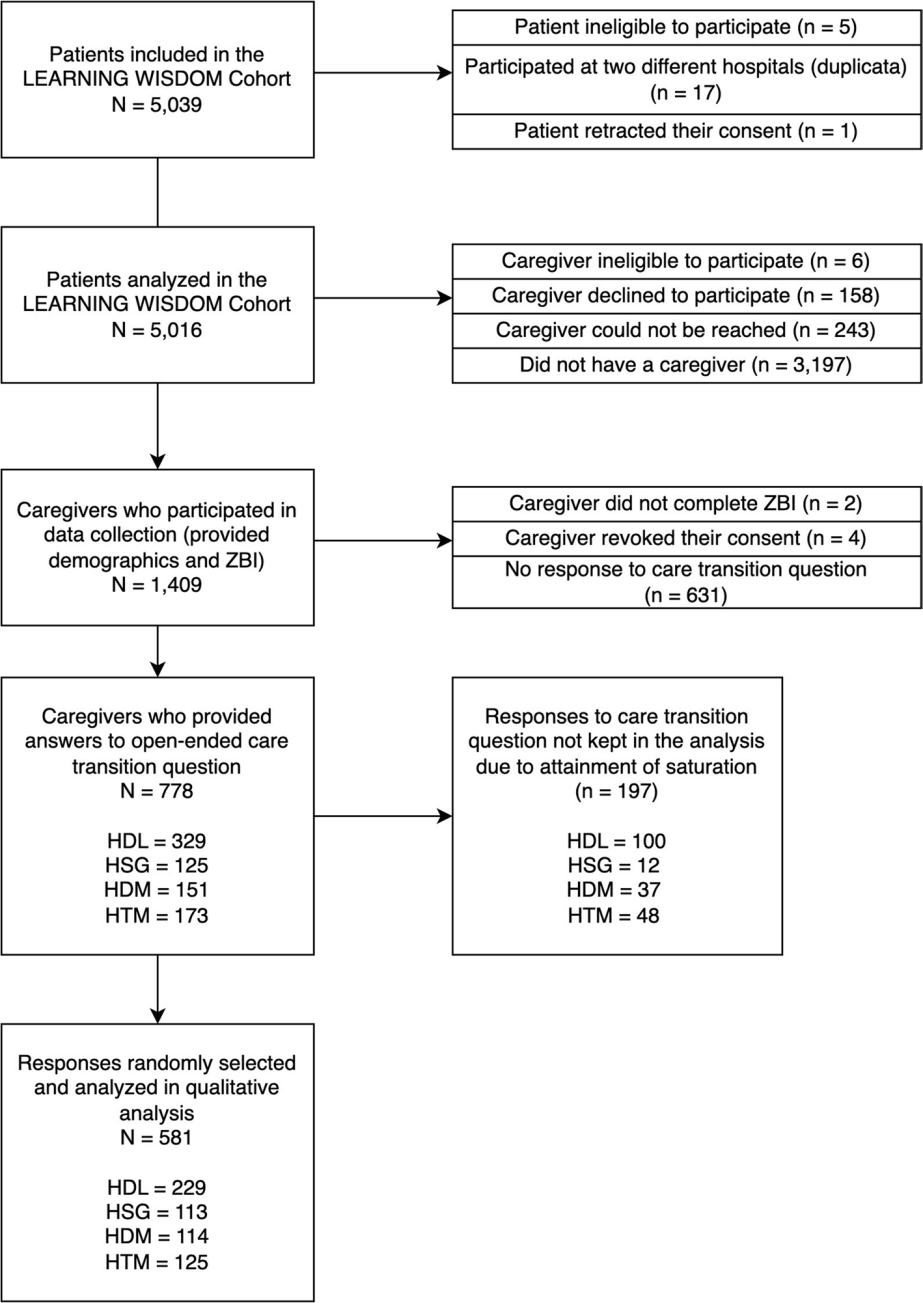
Flowchart describing the recruitment of patients and their caregivers

The total LEARNING WISDOM cohort included 5,016 participating patients (Fig. 1). Patients (*n* = 1819) allowed the research team to contact their caregiver, and 410 caregivers were excluded or declined to participate, leaving 1,409 patient-caregiver dyads. Of these, 778 caregivers provided open-response comments to Question A (Appendix A, French version). Of these caregivers, 752 responded to Question B.

Of 778 caregiver responses, 581 were analyzed, along with their ZBI questionnaires: 229 from HDL, 125 from HTM, 114 from HDM and 113 from HSG. Among caregivers, 138 (23.7%) reported an increase in caregiver burden following a care transition, 374 (64.3%) had an unchanged level of burden, 43 (7.4%) had an improved burden, and 26 (4.4%) opted not to comment. Demographic characteristics of 581 caregivers and patients are found in Table 1, stratified by self-reported change in burden. Among analyzed caregiver comments, 235 mentioned at least one sub-theme and 328 did not contain enough content to extract any themes. Concerning the overall emotional valence of these 253 comments, 60 were positive (23.7%), 33 neutral (13%), and 160 negative (63.2%).

**Table 1:** Demographic characteristics of patients and caregivers stratified by the caregiver’s self-reported change in burden

	**No comment** **(*N* = 26)** **4.4%**	**Burden increased** **(*N* = 138)** **23.7%**	**Burden unchanged** **(*N* = 374)** **64.3%**	**Burden improved** **(*N* = 43)** **7.4%**	**Overall** **(*N* = 581)**
**Hospital**					
HDL	10 (38.5%)	66 (47.8%)	137 (36.6%)	16 (37.2%)	229 (39.4%)
HSG	3 (11.5%)	21 (15.2%)	79 (21.1%)	10 (23.3%)	113 (19.4%)
HDM	10 (38.5%)	25 (18.1%)	72 (19.3%)	7 (16.3%)	114 (19.6%)
HTM	3 (11.5%)	26 (18.8%)	86 (23.0%)	10 (23.3%)	125 (21.5%)
**Patient age**					
Mean (SD)	80.1 (8.34)	78.4 (7.33)	76.5 (7.34)	77.7 (8.70)	77.2 (7.54)
**Patient gender**					
Man	12 (46.2%)	65 (47.1%)	198 (52.9%)	27 (62.8%)	302 (52.0%)
Woman	14 (53.8%)	73 (52.9%)	176 (47.1%)	16 (37.2%)	279 (48.0%)
**Arrival at the ED**					
Ambulance	12 (46.2%)	83 (60.1%)	184 (49.2%)	20 (46.5%)	299 (51.5%)
Walk-in	14 (53.8%)	55 (39.9%)	190 (50.8%)	23 (53.5%)	282 (48.5%)
**Canadian Triage Acuity Scale (CTAS)**					
1	0 (0%)	0 (0%)	1 (0.3%)	0 (0%)	1 (0.2%)
2	2 (7.7%)	16 (11.6%)	44 (11.8%)	9 (20.9%)	71 (12.2%)
3	17 (65.4%)	65 (47.1%)	198 (52.9%)	17 (39.5%)	297 (51.1%)
4	7 (26.9%)	51 (37.0%)	123 (32.9%)	13 (30.2%)	194 (33.4%)
5	0 (0%)	6 (4.3%)	8 (2.1%)	4 (9.3%)	18 (3.1%)
**Time on stretcher (hours)**					
Mean (SD)	10.3 (7.36)	12.1 (8.57)	10.5 (8.31)	14.3 (7.85)	11.2 (8.36)
**Charlson Comorbidity Index Score**					
Mean (SD)	6.12 (2.58)	5.04 (1.73)	5.06 (2.09)	5.42 (2.30)	5.13 (2.06)
**Covid-19 Wave at time of ED visit (Québec)**^**a**^					
Pre-pandemic	18 (69.2%)	107 (77.5%)	259 (69.3%)	26 (60.5%)	410 (70.6%)
Wave 1	7 (26.9%)	29 (21.0%)	111 (29.7%)	16 (37.2%)	163 (28.1%)
Between the end of wave 1 and wave 2	1 (3.8%)	2 (1.4%)	4 (1.1%)	1 (2.3%)	8 (1.4%)
**Have a family physician**					
Yes	24 (92.3%)	131 (94.9%)	352 (94.1%)	39 (90.7%)	546 (94.0%)
No	2 (7.7%)	7 (5.1%)	22 (5.9%)	4 (9.3%)	35 (6.0%)
**Can quickly get an appointment with family physician if needed**					
Yes	18 (69.2%)	77 (55.8%)	239 (63.9%)	28 (65.1%)	362 (62.3%)
No	8 (30.8%)	61 (44.2%)	135 (36.1%)	15 (34.9%)	219 (37.7%)
**Have access to transport**					
Yes	23 (88.5%)	127 (92.0%)	347 (92.8%)	41 (95.3%)	538 (92.6%)
No	3 (11.5%)	11 (8.0%)	27 (7.2%)	2 (4.7%)	43 (7.4%)
**People in social circle**					
Mean (SD)	4.31 (5.14)	3.30 (2.53)	4.07 (3.57)	3.72 (2.33)	3.87 (3.37)
**First language**					
French	26 (100%)	138 (100%)	373 (99.7%)	43 (100%)	580 (99.8%)
Missing	0 (0%)	0 (0%)	1 (0.3%)	0 (0%)	1 (0.2%)
**Patient ethnicity**					
Caucasian	26 (100%)	138 (100%)	374 (100%)	41 (95.3%)	579 (99.7%)
Missing	0 (0%)	0 (0%)	0 (0%)	2 (4.7%)	2 (0.3%)
**Patient highest level of education**					
Primary school	11 (42.3%)	60 (43.5%)	155 (41.4%)	20 (46.5%)	246 (42.3%)
Secondary school (DES)	6 (23.1%)	45 (32.6%)	96 (25.7%)	11 (25.6%)	158 (27.2%)
College (DEC)	4 (15.4%)	12 (8.7%)	40 (10.7%)	3 (7.0%)	59 (10.2%)
Vocational studies (DEP)	2 (7.7%)	11 (8.0%)	34 (9.1%)	3 (7.0%)	50 (8.6%)
University studies	3 (11.15%)	9 (5.5%)	49 (13.1%)	6 (14%)	67 (11.15%)
Missing	0 (0%)	1 (0.7%)	0 (0%)	0 (0%)	1 (0.2%)
**Patient income**					
Less than 10 000$	0 (0%)	5 (3.6%)	10 (2.7%)	4 (9.3%)	19 (3.3%)
10 000 to 19 999$	6 (23.1%)	40 (29.0%)	64 (17.1%)	9 (20.9%)	119 (20.5%)
20 000 to 29 999$	5 (19.2%)	34 (24.6%)	65 (17.4%)	5 (11.6%)	109 (18.8%)
30 000 to 39 999$	3 (11.5%)	13 (9.4%)	43 (11.5%)	3 (7.0%)	62 (10.7%)
40 000 to 49 999$	5 (19.2%)	5 (3.6%)	34 (9.1%)	2 (4.7%)	46 (7.9%)
50 000 to 59 999$	1 (3.8%)	1 (0.7%)	11 (2.9%)	2 (4.7%)	15 (2.6%)
60 000 to 69 999$	1 (3.8%)	2 (1.4%)	8 (2.1%)	2 (4.7%)	13 (2.2%)
70 000 to 79 999$	0 (0%)	1 (0.7%)	6 (1.6%)	1 (2.3%)	8 (1.4%)
80 000 to 89 999$	0 (0%)	2 (1.4%)	1 (0.3%)	1 (2.3%)	4 (0.7%)
90 000 to 99 999$	0 (0%)	0 (0%)	2 (0.5%)	0 (0%)	2 (0.3%)
More than 100 000$	0 (0%)	2 (1.4%)	11 (2.9%)	1 (2.3%)	14 (2.4%)
Missing	5 (19.2%)	33 (23.9%)	119 (31.8%)	13 (30.2%)	170 (29.3%)
**Patient housing type**					
Home alone, intermediate or family-type residences, or public housing	5 (11.5%)	43 (29.0%)	70 (18.2%)	14 (32.6%)	132 (21.5%)
Home, shared with a spouse or family	19 (73.1%)	80 (58.0%)	267 (71.4%)	25 (58.1%)	391 (67.3%)
Retirement home	2 (7.7%)	15 (10.9%)	37 (9.9%)	4 (9.3%)	58 (10.0%)
**Patient-Caregiver relationship**					
Friend, sibling, or other	4 (15.4%)	11 (8.0%)	45 (12.0%)	6 (14.0%)	66 (11.4%)
Parent–Child	9 (34.6%)	64 (46.4%)	116 (31.0%)	19 (44.2%)	208 (35.8%)
Spouse	13 (50.0%)	63 (45.7%)	212 (56.7%)	18 (41.9%)	306 (52.7%)
Missing	0 (0%)	0 (0%)	1 (0.3%)	0 (0%)	1 (0.2%)
**Caregiver age**					
Mean (SD)	64.7 (11.2)	62.8 (13.2)	65.4 (11.8)	62.0 (13.7)	64.5 (12.3)
**Caregiver gender**					
Man	3 (11.5%)	45 (32.6%)	118 (31.6%)	10 (23.3%)	176 (30.3%)
Woman	23 (88.5%)	93 (67.4%)	256 (68.4%)	33 (76.7%)	405 (69.7%)
**Caregiver first language**					
French	26 (100%)	137 (99.3%)	370 (98.7%)	43 (100%)	576 (99.0%)
English	0 (0%)	1 (0.7%)	4 (1.1%)	0 (0%)	5 (0.9%)
**Caregiver highest level of education**					
Primary school	9 (34.6%)	56 (40.6%)	142 (38.0%)	18 (41.9%)	225 (38.7%)
Secondary school (DES)	6 (23.1%)	45 (32.6%)	98 (26.2%)	11 (25.6%)	160 (27.5%)
College (DEC)	7 (26.9%)	17 (12.3%)	64 (17.1%)	8 (18.6%)	96 (16.5%)
Vocational studies (DEP)	3 (11.5%)	12 (8.7%)	36 (9.6%)	3 (7.0%)	54 (9.3%)
University studies	1 (3.8%)	8 (5.8%)	34 (9.1%)	3 (7.0%)	46 (7.9%)
Graduate school	0 (0%)	0 (0%)	0 (0%)	0 (0%)	0 (0%)
**Caregiver income**					
Less than 10 000$	1 (3.8%)	6 (4.3%)	8 (2.1%)	2 (4.7%)	17 (2.9%)
10 000 to 19 999$	3 (11.5%)	19 (13.8%)	25 (6.7%)	4 (9.3%)	51 (8.8%)
20 000 to 29 999$	4 (15.4%)	11 (8.0%)	42 (11.2%)	6 (14.0%)	63 (10.8%)
30 000 to 39 999$	2 (7.7%)	11 (8.0%)	43 (11.5%)	9 (20.9%)	65 (11.2%)
40 000 to 49 999$	4 (15.4%)	18 (13.0%)	35 (9.4%)	3 (7.0%)	60 (10.3%)
50 000 to 59 999$	1 (3.8%)	8 (5.8%)	18 (4.8%)	3 (7.0%)	30 (5.2%)
60 000 to 69 999$	0 (0%)	4 (2.9%)	22 (5.9%)	0 (0%)	26 (4.5%)
70 000 to 79 999$	3 (11.5%)	3 (2.2%)	9 (2.4%)	1 (2.3%)	16 (2.8%)
80 000 to 89 999$	0 (0%)	2 (1.4%)	12 (3.2%)	1 (2.3%)	15 (2.6%)
90 000 to 99 999$	0 (0%)	2 (1.4%)	9 (2.4%)	0 (0%)	11 (1.9%)
More than 100 000$	1 (3.8%)	16 (11.6%)	25 (6.7%)	4 (9.3%)	46 (7.9%)
Missing	7 (26.9%)	38 (27.5%)	126 (33.7%)	10 (23.3%)	181 (31.2%)
**ZBI score**					
Mean (SD)	5.73 (5.42)	10.5 (7.87)	6.57 (7.02)	7.65 (7.21)	7.55 (7.36)
**Delay between ED index visit and caregiver recruitment (days)**					
Mean (SD)	24.8 (15.8)	22.9 (19.1)	27.9 (33.0)	27.1 (17.4)	26.5 (28.7)

^a^Dates from the *Institut national de santé publique du Québec* (INSPQ): Pre-pandemic (Before March 13th, 2020), wave 1 (Between March 13th, 2020, to 11th of July 2020), Between the end of wave 1 and the beginning of wave 2 (12th of July 2020 to the 22nd of August 2020)

## Quantitative results

The mean score on the ZBI among all caregivers included in the qualitative analysis (*N* = 581) was 7.55 (SD = 7.36), with a median of 5 (IQR = 2–11; Range = 0–38). The internal consistency was high (α = 0.879, 95% CI = [0.86, 0.89]).

### Changes in caregiver burden following a visit to the emergency department

Cohen’s Kappa was calculated to quantify interrater reliability between the two coders (κ = 0.989, 647 comments). Results of the ANOVA analysis revealed statistically significant differences in self-reported burden across the four categories (Binomial test χ^2^ = 530.5, *p* < 0.001): most caregivers reported that their level of burden did not change (64.3%), but 23.8% of caregivers reported an increase in caregiver burden.

### Changes in caregiver burden following a visit to the emergency department and scores on the ZBI

The effect of subjective change in burden on ZBI score was statistically significant (*F*(3, 191.62) = 11.83, *p* < 0.001). Caregivers who chose not to comment had the lowest ZBI scores (*n* = 26, *M* = 5.73, *SD* = 5.14), followed by caregivers with a level of burden left unchanged (*n* = 374, *M* = 6.56, *SD* = 7.02). Next were caregivers with a level of burden that improved following the patient's visit to the emergency department (*n* = 43, *M* = 7.65, *SD* = 7.21). Caregivers who reported an increase in their subjective burden had the highest ZBI scores (*n* = 138, *M* = 10.53, *SD* = 7.87). ZBI scores among the increased burden group were statistically significantly different from the unchanged burden group (*t*(510) = 5.54, *p* < 0.001, *d* = 0.55), and the no comment group (*t*(162) = 3.12, *p* = 0.01, *d* = 0.66).

There was a statistically significant effect of change in burden on ZBI score even when controlling for age and comorbidities (*F*(3, 575) = 11.03, *p* < 0.001), and there was a statistically significant effect of Charlson Comorbidity Index score on ZBI score (*F*(1, 575) = 4.95, *p* = 0.026) but there was no effect of patient age on ZBI score (*F*(1, 575) = 0.007, *p* = 0.935).

## Qualitative results

### Analysis of themes within the content analysis

**Fig. 2:**
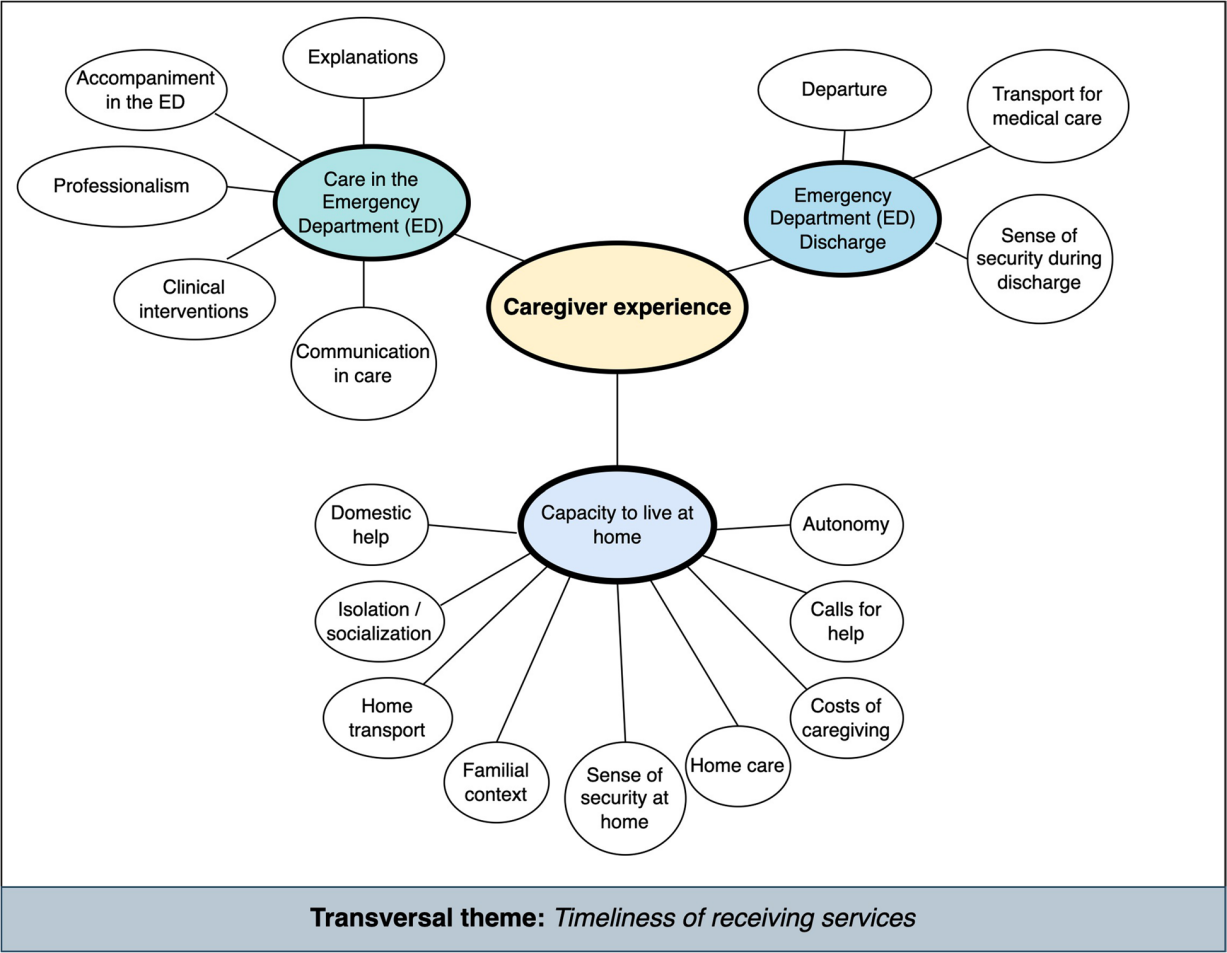
Mind map of the three main themes and seventeen sub-themes emerging from caregivers’ responses to the question: “In your opinion, what could be improved to reduce the burden of care for [the patient]?” following the patient’s transition of care from the emergency department to home. A transversal theme, *Timeliness of receiving services* is also identified. See Fig. 3 for frequencies of each sub-theme mentioned and a graphical representation of the relative proportion of emotional valence associated with each sub-theme

**Fig. 3:**
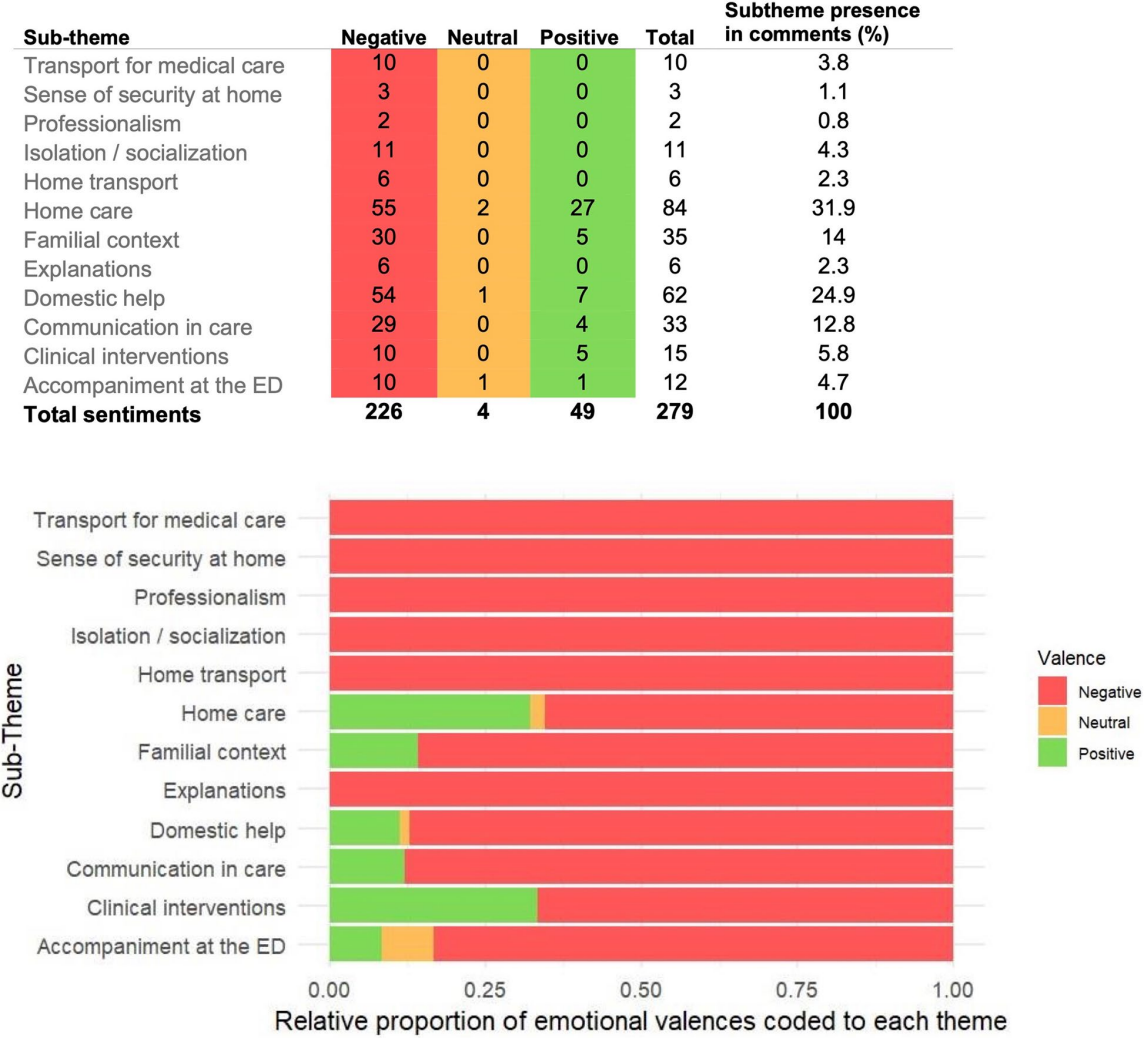
**A** Frequency of sub-themes, stratified by emotional valence emerging from 253 caregiver comments. **B** Relative proportions of negative, neutral, and positive sentiments coded to each theme embedded in 253 caregiver comments. For both A and B, red represents negative theme mentions, yellow neutral, and green positive

Our final caregiver-centered care transition coding scheme contained three main themes: *Care in the emergency department*, *Emergency Department Discharge* and *Capacity to live at home* and 14 sub-themes (Fig. 2). See Appendix D for a definition of each theme, sub-theme, and an example of each. We counted when caregivers specifically mentioned each of three statements: an explicit call for help or information, a mention of time or financial costs associated with caregiving, and a mention of their patient’s autonomy for a total of 17 sub-themes (Fig. 3). After making these amendments, 50 randomly selected comments were analyzed per coder, and again inter-rater reliability was very high (κ = 0.991).

### Care in the emergency department

Both comments citing *Professionalism* in the emergency department were negative. One caregiver felt the personnel at the ED lacked humanity and understanding of the situation. Another caregiver felt their physician was being negligent in their duties, changing the patient’s prescriptions without consulting the patient nor the caregiver.

*“There is a lack of consistency among physicians. One played around with [the patient’s] prescriptions, decreased her medication dose without even examining her, without even looking at her back problem. [The physician] said “cut this” and I am the one who sees the repercussions of this […] I would like to have something to give her to relieve her pain. Pain is the worst thing. It’s* *as* *hard for me as it is for her.”* [C318, HDL]

*Explanations* were viewed as negative by 6 caregivers. Two caregivers mentioned a lack of instruction for managing the patient’s conditions post discharge. One caregiver mentioned that they would have preferred to speak to the physician themselves because they felt as though the patient was withholding information and was being stubborn about adherence to treatment. Two caregivers mentioned having to relay information to the patient, who did not understand the information given to them by personnel.

*“It’s difficult because I have a stubborn mother, and I don’t get to see the doctors myself. More complete explanations to help my mother would be appreciated because I feel like she’s minimizing the problem to protect me. I’d like to make sure I help her properly.”* [C330, HDL]

Regarding *Communication in care*, 5 caregivers reported successful follow-up between departments or with specialists following their departure from the ED. Among negative commenters, 3 reported that they were still waiting to see a specialist, 8 mentioned gaps in access to information or wanting to know where and how to request different services. Four specifically suggested telephone follow-up calls to pass along updates and to see how patients are managing at home. Four specifically mentioned difficulty reaching their family physician, and the remaining comments referred to waiting for the ED to transfer information or requests to other departments or outside services like convalescence homes, social services, and specialists.

*“[…] It is a chore to be able to open a medical file at the [local community service center]. Getting access to resources is not as bad [after a visit to the ED], but this process would really need to be reviewed. It’s long and painful.”* [C507, HDM]

Caregivers who reported *Accompaniment* of the patient to the ED tended to view this theme negatively. Five caregivers mentioned assistance with or having someone else accompany the patient to their appointments would be helpful. Four caregivers mentioned that accompanying the patient was personally taxing or affected their work schedule. One caregiver described relief that ten months of treatment requiring weekly visits was coming to an end. One caregiver reported positively, and one neutrally that they accompanied the patient to their medical appointments following an initial visit to the ED. One caregiver reported frustration that despite being there during the appointment, she was not allowed into the doctor’s office and that her mother would forget what the doctor had told her.

*“[We would like] more accompaniment and follow-up outside the [emergency department]. This could prevent him from returning so often for the same thing.”* [C94, HDL]

Of the 15 caregivers citing *Clinical intervention*s in their comments, 7 mentioned the quality of care;, of which 2 were negative. One caregiver felt the management of the patient’s condition had room for improvement, while another caregiver felt that the patient was being treated with medications that only made them sicker, saying “*They just give her pills. She overdoses and then they give her other pills*” [C404, Hospital B]. The remaining comments cited care at the ED as good or excellent. Three other sub-themes emerged, and all were negatively referenced. One caregiver cited an error with medications, 3 felt the waiting time at the ED was too long, and one caregiver felt the patient was not given the correct diagnosis, saying “…*that he would have been properly diagnosed, I would have liked him to be seen and re-evaluated in geriatrics*” [C172, HSG].

### Capacity to live at home

This main theme, related to services empowering older adults to stay independent at home, was dominated by comments about *Home care*. We coded comments as negative if a service was requested but not yet delivered, in addition to negative experiences with homecare services that were delivered. Of these, 37 (22% positive) mentioned requesting or receiving care from a local community service center (or also known as “CLSC” in Québec), 10 (40% positive) mentioned receiving or requesting home visits from a physician or nurse.

*“Blood tests [could be done] at home, getting up [to get to there] is difficult, [and] it’s too long to wait five hours in the morning without having eaten.”* [C530, HTM]

Nineteen mentioned living in or requesting adapted living environments (74% positive), and 8 mentioned they were waiting to be assigned a family physician; all of which were negative.

*“[Would] appreciate having a family doctor. We’ve been on the waiting list for two years. It’s worrying.”* [C38, HDL]

Eleven caregivers mentioned *Isolation* as an area for improvement. Three mentioned wishing the patient lived closer, and the remainder mentioned wanting someone (a volunteer, other family member, or guardian) to spend some time with the patient, either to help with loneliness or boredom, or to accompany them to activities outside the home.

*“One big problem is isolation. He suffers from boredom and loneliness. It’s a vicious circle [because the] burden becomes even heavier. I feel that we are his only source of happiness.”* [C403, HDM]

Similarly, *Home Transport* was used to code 6 mentions of non-medical transport. All comments requested access to transport for everyday needs like grocery shopping, and two caregivers suggested that access to this kind of transport would allow the patient to independently run errands.

*“[I would like to see] an increase in people able to meet the needs of everyday life, like transport for errands.”* [C124, HDL].

The *Familial context* emerged 35 times in the dataset, with most comments negative (86%). Two caregivers mentioned they did not mind taking care of their spouses, seeing it as part of their role. Three others mentioned other family members pitched in to help, dividing the workload. One of these caregivers mentioned ongoing discussions between their mother and the rest of the family, trying to convince their mother to sell the family home and move to a more manageable residence. Similar discussions were reported negatively, with 4 caregivers mentioning the main problem was convincing the patient to accept their condition or to accept help.

*"My mother is taking care of my father [who has Alzheimer’s] and I wish someone would take care of my father so that my mother can take care of herself. My mother has anxiety attacks. She can't have help from the [local community service center] because my father does not want to see anyone. If he does not accept care, my mother does not accept it either. I am less and less confident in her [abilities to take care of him]. I’m tired of always trying to convince my mother [to accept help]."* [C179, HDM]

Five caregivers expressed frustration that they were not involved in shared decision-making about care and would have liked to be part of that process. The remainder expressed conflict or frustration with other family members, usually a desire for other family members to pull their weight or visit the patient from time to time. One caregiver mentioned wishing her son could trust that she was able to adequately care for his father.

Concerning *Domestic help*, seven positive comments mentioned that the caregiver felt housekeeping or cooking duties were adequately addressed. Caregivers with negative comments (54) mentioned requesting help with household cleaning, making meals, and shoveling snow. Two caregivers mentioned a service cooperative handled these tasks but canceled their services during the pandemic.

*“[…] get some outside help. For example, it was snowing this week, but [the patient] cannot shovel, so I had to drive 20 km to clear his driveway. Help with housekeeping would be nice too.”* [C24, HDL]

For three exploratory themes, *Calls for help or information* occurred 27 times, with caregivers requesting help for household tasks that they were not comfortable with (e.g., toileting, bathing). Most of these comments requested more services to help with respite, and to understand which services are available and how to request them through proper channels. One caregiver mentioned having information on how to “*get through it (or cope) when the situation becomes complicated*” [C297, Hospital A] would be helpful. Among comments citing the *Costs of caregiving*, 8 mentioned financial costs, with 2 caregivers mentioning that they would appreciate government financial aid. One caregiver mentioned an accumulation of stress that she felt was *costing* her own wellbeing. Caregivers who mentioned financial costs also often cited costs to time (4), mentioning taking time off work, or working less to fulfill their caregiving role.

*“[I’d suggest to] maybe pair the appointments so they’re all on the same day. I’d miss less work.”* [C108, HDL]

*Autonomy* was another common mention, occurring 35 times. In 28 cases, caregivers mentioned the patient was autonomous, and in these cases, caregivers reported overall positively. In 24 cases, there was nothing else to add in response. That is, autonomy was the only theme mentioned, and often in a one-word answer: *“autonomous”*.

*“[I have nothing to add] he is one hundred percent autonomous!”* [C147, HDM]

One caregiver reported that the patient was not autonomous, and that she dared not leave him alone. Another reported that the patient was living at home alone but suspected that they needed help with household tasks but would not ask for them.

### Departure

We considered mentions of medical *Transport* to fall under a larger theme of *Departure* but all mentioned medical transport specifically: including transport to medical appointments and procedures (7 comments) or mentions that adapted transport should be improved to allow older patients to autonomously attend their own appointments (3). All these comments mentioned gaps in the availability of transport, so they were coded negatively.

*“Transportation for medical appointments. When she goes for blood transfusions, it’s long. It would be nice if someone else could do it [handle transportation].”* [C533, HTM]

### Timeliness of services in and after the ED

The timeliness of receiving services in and after ED care was a transversal theme. We did not identify the timeliness of services as an individual sub-theme as it affected each major theme individually. For *Care in the emergency department*, 3 caregivers felt their wait time at the ED was too long, 3 caregivers mentioned long wait times to get follow-up care with a specialist after the ED visit, and 5 were still waiting for the ED to transfer information to a third party. For *Capacity to live at home*, issues with timeliness of services were most apparent, as we coded comments as negative if a service was requested but not yet delivered.

*“[We are] waiting for [local community service center] care, [and there is a] lack of follow-up between hospital and rehabilitation. It's very long.”* [C367, HDL]

The most frequent unmet requests for service were regarding awaiting services from local community service centers (CLSCs; 29 mentions, including home care services specifically; 6 mentions), and waiting to be assigned a family physician (8 mentions). All comments mentioning *Discharge* were coded negatively: highlighting gaps in the availability of timely and convenient transport.

### Caregivers who did not mention any theme

Of 331 caregivers who did not mention any themes in their comments, (85.7%) were neutral in tone, citing that they could not think of anything to report, or that they did not experience any burden of care to speak of. Forty-three caregivers mentioned that they had nothing to report because things were going well. One caregiver mentioned that things were going well and that they were content with the resources at their disposal. Another caregiver mentioned that everything was fine because she was retired and able to meet the patient’s needs, but acknowledged that if she had still been working, things would have been different. Only four caregivers left negative comments with no main themes emerging. One caregiver mentioned displeasure with their role as a caregiver, and another caregiver mentioned wishing that the patient would be able to function on their own. Another voiced that they could not think of anything that would help the patient, and a final comment mentioned the patient was in pain, but nothing could be done except to wait for the pain to pass.

### Co-occurrence networks

**Fig. 4:**
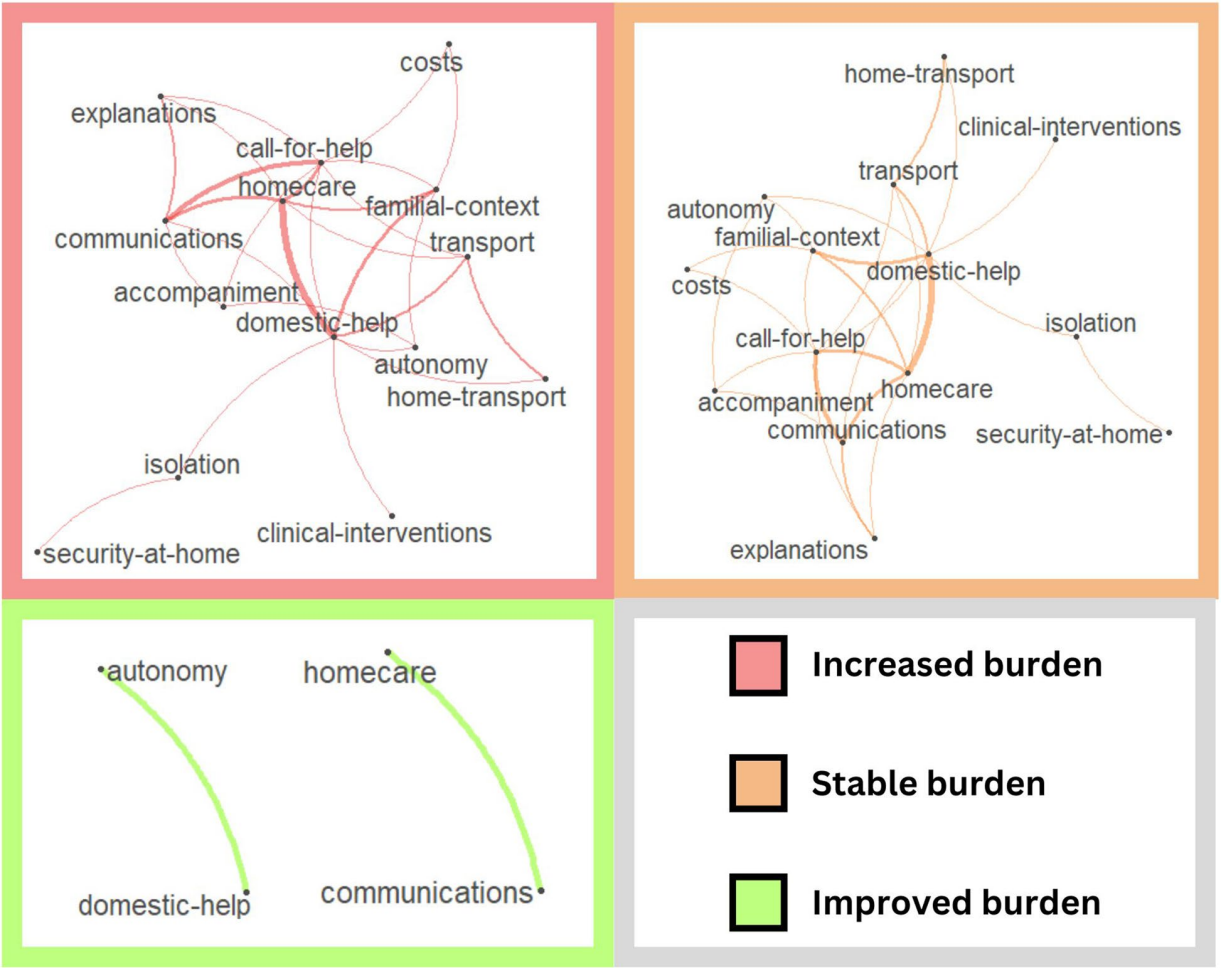
Co-occurrence networks of themes appearing in comments, by self-reported level of burden following departure from the emergency department. Lines between themes indicate co-occurrences within the same comment. Thinner lines represent fewer co-occurrences between two themes, and thicker lines denote more frequent co-occurrences

Following the analysis of themes and sub-themes, we noticed that some caregivers gave comments containing more than one theme (*n* = 71). We stratified the dataset by self-reported change in burden (*increased* = 30, *stable* = 28, *improved* = 2; 11 caregivers did respond regarding a self-reported change in burden) to visualize which themes occur together and how they interact on this basis (Fig. 4).

For caregivers reporting an unchanged (stable) burden following an ED care transition, central interconnected themes include communications, calls for help, home care, and domestic help. For caregivers reporting an increased burden, interconnected themes appear much more complex and include accompaniment, the familial context, costs, domestic help, explanations, calls for help, and communications. The bands linking domestic help to the familial context, costs, and calls for help are also thicker, indicating a greater importance of these themes in the comments of caregivers reporting an increase in subjective burden. For caregivers reporting a reduced burden, only two links emerged: the link between autonomy and domestic help, and homecare and communications. These links also emerge in the stable and increased burden groups, indicating that these two joined sets of themes may be important for all the caregivers we surveyed.

## Discussion

We conducted a mixed methods design to understand, from the caregiver’s point of view, A) changes in burden of care following transitioning a patient’s care from the ED and B) what can be improved in the patient’s transition of care in this context. We also leveraged the French version of the Zarit Brief Burden interview (ZBI-12) to corroborate scores on caregiver burden with caregiving realities as reported by caregivers.

Changes in subjective burden appeared to correspond with ZBI scores. Greater ZBI scores are given to mean a greater level of caregiver burden. Most caregivers reported that their level of burden did not change (64.3%), and their average ZBI score was 6.57 on 48, considered a moderate level of burden [9]. Caregivers reporting an increase in burden (23.8%) had an average ZBI of 10.5, considered high burden [9]. Mean scores of caregivers who reported an improvement in subjective burden (7.4%) were 7.65. Only the ZBI scores of the caregivers experiencing an increase in burden statistically differed from caregivers with stable burden. This effect remained when controlling for age and comorbidities of the patient. In the original French-Canadian ZBI-12 validated with caregivers of the patient with dementia, scores between 3 and 8 indicated moderate burden and scores greater than 18, severe burden [9]. The cut-point signaling significant caregiver burden in other populations can be as low as 11 [36], 12 [37], 13 [38], up to 17 [39, 40]. This ZBI cutoff score appears to increase as the patient’s cognitive function decreases [40].

Caregivers who have higher levels of burden likely have greater room for improvements to burden, which may explain why caregivers in this improved burden group have higher ZBI scores than caregivers in the stable burden group. This effect has been documented in interventional studies aiming to reduce caregiver burden: caregivers with higher baseline burden experienced the greatest benefits in reducing burden [41]. Without baseline questionnaire data, we are limited to speculation as to how ZBI scores were affected by the care transition from the ED.

We also extracted themes from caregiver comments to triangulate experiences in caregiver burden with ZBI scores. Most caregivers did not mention any themes in their comments, most neutrally citing they had nothing to report regarding what could be changed to improve their experiences of burden. ZBI scores of caregivers reporting no change in burden following the ED care transition were the lowest, and accordingly, caregivers with higher ZBI scores corresponded to reporting an increase in burden following an ED care transition. Those caregivers reported greater needs in homecare, domestic help, and greater challenges in communications and follow-up, the familial context, and in calls for help.

Caregivers providing comments reported concepts negatively, either indicating dissatisfaction or not yet receiving a service. *Home care* was the most prevalent sub-theme, with both positive and negative experiences discussed, followed by *Domestic help* and *Familial context*. Within home care related comments, outpatient care from community clinics and home visits from a physician or nurse were appreciated by caregivers.

Communications and follow-up were also highlighted, mostly negatively. This theme has been negatively cited elsewhere in qualitative research of caregiver experiences in a transition from acute to community care [21, 42, 43]. This suggests that currently, there are serious inadequacies in communication between care settings, and therefore threats to the continuity of care for patients and their caregivers following emergency treatment. Issues with the timeliness of services for home care were more pronounced in caregiver comments than by the patients in this same cohort [21]. Involving caregivers either by calling them from the ED or including them in the construction of discharge plans as crucial to the feasibility and therefore the success of care transitions.

*Clinical interventions* were mentioned, with mixed sentiments, while *Accompaniment and Isolation* were predominantly viewed negatively. *Home Transport* and *Capacity to live at home* were discussed often with negative connotations. In a previous analysis of themes emerging from patients in the care of these caregivers [21], patients were both more frequent and more positive reporters on the quality of clinical interventions. This illustrates important differences between patient and caregiver perspectives within the same dyad. *Domestic help* was the most common sub-theme among caregivers, but *Communications and follow-up* were similarly referenced frequently as an area for improvement among both patients and their caregivers. If follow-up could be appropriately managed with the involvement of primary care, integrated case management [44] facilitated by access to a single electronic medical record [45], and better communication of goals of care [46], time and energy caregivers spend coordinating care could be spent on other affairs, including domestic help, navigating familial responsibilities, or simply passing quality time with the patient.

We split the dataset based on caregivers' self-reported changes in burden (whether it increased, remained stable, or improved) to visually depict the co-occurrence and interactions of various sub-themes. Among caregivers who indicated a stable level of burden after an ED care transition, key interrelated sub-themes were *Communications*, *Calls for help*, *Home care*, and *Domestic help*. In cases of increased burden, the interconnections among sub-themes were more complex, and connections linking domestic help to family context, finances, and calls for help were more pronounced, indicating their heightened importance to this group and greater needs of highly burdened caregivers. Caregivers report an improved burden only on *Autonomy* and *Domestic help*, and *Home care* and *Communications*, suggesting that these sub-themes are common experiences across the spectrum of burden. Caregivers who call for help or those facing the declining autonomy of a patient could benefit from care navigators or public awareness campaigns to present the services available to them in their communities (e.g. respite care for caregivers), and their own rights and protections (e.g. employment or familial status) under local laws.

### Strengths and limitations

The strengths of our study arise from the application of both quantitative and qualitative methodologies, and from substantial random sampling at four different EDs. Our strong inter-rater reliability indicates a clear coding scheme, which we attribute to the iterative hybrid development of an original coding scheme developed for patient comments.

Limitations of this study include the short nature of responses from caregivers. Open-response data can lack data substantial enough to achieve substantial credibility and resonance [47]. However, we were able to analyze several hundred comments—one way to boost the richness of otherwise sparse data. We acknowledge that an important proportion did not comment on their burden level (4.5% replied “no comment” to Question A). The fact that caregivers took the time to complete the ZBI during this phone call, and provided generally short responses about improvements to care transitions might be a reflection of these caregivers truly not feeling or knowing what can be improved (48.7% of caregivers said they had nothing to report or nothing they feel could be improved in response to Question B) and not a result of insufficient sampling. We were also not able to distinguish between incident (sudden) and long-term caregivers [48] or describe caregivers’ own health status or comorbidities [49]. Both factors likely impact both caregiver burden and the quality of care transitions, which presents an avenue for future research.

Another limitation is that comments for this study were collected over the telephone and transcribed immediately by a research professional. We did not audio record caregivers’ comments. This may have introduced an information bias such that the content was filtered by the research professional conducting the interviews. Follow-up time poses another challenge, as patients were called to participate between 1–7 days following discharge. Follow-up time for caregivers (*M* = 26 days) was much more variable than the weeklong follow-up time of patients. Collection soon after ED discharge may capture the caregiver’s experience but if too soon, may not have left sufficient time to undergo all relevant aspects of the care transition.

Overall, our study highlights the importance of screening for caregiver burden in the ED. Better understanding caregiver unmet needs can guide ED clinicians in finding adapted solutions to meet these needs. While it may seem that discussions of caregiver burden may prove prohibitively time-consuming, shorter and swifter tools have evidence of efficacy in older adult populations. One national American study found that three-quarters of primary care physicians felt responsible to identify caregiver needs when seeing patients, and half felt it important to address caregiver health and mental health in their assessment [50]. Physicians were four times as likely to take caregiver needs into consideration if they themselves acted in a caregiving role [50]. We are optimistic that while caregivers often cited gaps in home healthcare services, physicians and decision-makers appear to be open to screening for caregiver burden at the ED and to integrate caregiver needs into care transition plans, especially as caregiving is becoming more common.

## Conclusion

We used a mixed methods approach to understand the caregiver's perspective regarding caregiver burden following a patient’s transition from the ED to home. Only caregivers facing an increased self-reported burden showed significantly different ZBI scores compared to those with stable burden levels, which persisted even when accounting for patient age and comorbidities. Caregivers with greater initial burden may benefit most from targeted interventions designed to support caregiving. Screening for high caregiver burden or burnout at the ED and involving caregivers in the goals and trajectory of care can both inform tailored interventions aimed at reducing individual caregiver burden and enhance system-level policies that target improved care transition processes. The most salient targets for reform are the inadequacy in communications and follow-up between healthcare settings along the care continuum, and improving access to homecare and domestic help. Addressing these unmet needs may improve the wellbeing of both caregivers and those in their care following emergency treatment.

## Supplementary Information

Supplementary Material 1.

## Data Availability

Anonymized data that support the findings of this study are available on request from the corresponding author, [NG]. The data are not publicly available as to protect the privacy of research participants.
